# 5-Fluorouracil resistant colon cancer cells are addicted to OXPHOS to survive and enhance stem-like traits

**DOI:** 10.18632/oncotarget.5991

**Published:** 2015-10-21

**Authors:** Corti Denise, Paolo Paoli, Maura Calvani, Maria Letizia Taddei, Elisa Giannoni, Scott Kopetz, Syed Mohammad Ali Kazmi, Morelli Maria Pia, Piergiorgio Pettazzoni, Elena Sacco, Anna Caselli, Marco Vanoni, Matteo Landriscina, Paolo Cirri, Paola Chiarugi

**Affiliations:** ^1^ Department of Experimental and Clinical Biomedical Sciences, University of Florence, Florence, Italy; ^2^ Department of Genomic Medicine, The University of Texas MD Anderson Cancer Center, Houston, Texas, USA; ^3^ SYSBIO Centre for Systems Biology, Department of Biotechnology and Biosciences, University of Milano-Bicocca, Milano, Italy; ^4^ Medical Oncology Unit, Department of Medical and Surgical Sciences, University of Foggia, Foggia, Italy

**Keywords:** cancer metabolism, OXPHOS, chemoresistance, metformin, cancer stem cells

## Abstract

Despite marked tumor shrinkage after 5-FU treatment, the frequency of colon cancer relapse indicates that a fraction of tumor cells survives treatment causing tumor recurrence. The majority of cancer cells divert metabolites into anabolic pathways through Warburg behavior giving an advantage in terms of tumor growth. Here, we report that treatment of colon cancer cell with 5-FU selects for cells with mesenchymal stem-like properties that undergo a metabolic reprogramming resulting in addiction to OXPHOS to meet energy demands. 5-FU treatment-resistant cells show a *de novo* expression of pyruvate kinase M1 (PKM1) and repression of PKM2, correlating with repression of the pentose phosphate pathway, decrease in NADPH level and in antioxidant defenses, promoting PKM2 oxidation and acquisition of stem-like phenotype. Response to 5-FU in a xenotransplantation model of human colon cancer confirms activation of mitochondrial function. Combined treatment with 5-FU and a pharmacological inhibitor of OXPHOS abolished the spherogenic potential of colon cancer cells and diminished the expression of stem-like markers. These findings suggest that inhibition of OXPHOS in combination with 5-FU is a rational combination strategy to achieve durable treatment response in colon cancer.

## INTRODUCTION

Colon cancer is one the most common tumors in western countries. Together with surgical intervention and radiotherapy, chemotherapy represents the first line of intervention to combat expansion of the tumor mass. Even with a promising initial response to pharmacological therapy, the majority of patients do not realize complete eradication of tumor cells, mainly due to selection of chemo-resistant cancer cells (CCCs). CCCs are a sub-population of tumor cells refractory to conventional drugs or radiotherapy and highly prone to metastasize [1; 2]. For this reason, the presence of CCCs is often associated with a poor prognosis [3]. Indeed, overcoming chemo- resistance is one of the major challenges in oncology.

Despite an active and growing interest studying CCCs, the origin of CCCs and the phenotype that defines them remains strongly debated [4]. It has been suggested that CCCs could derive from cancer stem cells (CSCs), a non-differentiated subset of cancer cells isolated from several different tumors and characterized by an intrinsic resistance to apoptosis as well as by unusual phenotypic plasticity [5]. Some evidence suggests that drug-resistant stem-like cells could also originate from other cancer cells when environmental conditions become unfavorable [6]. According to this model, under stresses such as hypoxia/anoxia, extreme acidity, nutrient deprivation, or contact with particular stromal cells, specific cancer cells can enter a slow proliferating/quiescent state, thus avoiding apoptosis [7–9]. When environmental conditions become permissive, these surviving cells re-differentiate, restarting to proliferate generating a heterogeneous tumor.

Several lines of evidence demonstrate that CCCs behave as CSCs, thus suggesting a common origin [10]. In fact most CCCs demonstrate a stem-like immunophenotype, self-renewal and tumor initiating capacity, as well as high motility and resistance to apoptosis. In addition, these cells have been shown to proliferate and regenerate new tumor masses in several mouse models [9]. These data suggest that CCCs resemble CSCs and are likely characterized by a peculiar metabolism, allowing them to survive stress conditions, and likely also contributing to their resistance to cytotoxic treatments.

Many highly proliferative cancer cells rely mainly on aerobic glycolysis (Warburg metabolism) for their energetics, which exploits carbons from glucose to produce ATP and other intermediates useful to sustain rapid growth [11; 12]. However, this metabolic strategy is not the most efficient to sustain a slow proliferating/quiescent state [13]. Indeed, recent evidence supports the hypothesis that acquired resistance to therapy is accompanied by a metabolic shift toward respiratory metabolism [14; 15], suggesting that metabolic plasticity can have a role in survival of cells responsible for tumor relapse. For example, it has been observed that several drug-resistant tumor cells show a higher respiratory activity than parental cells [16–18]. In addition, different studies suggested that metformin, an inhibitor of complex I of electron transport chain, preferentially kills drug-resistant cancer cells derived from different tumors, but has no effect on parental cells, reinforcing the hypothesis that acquisition of drug resistance may be accompanied by a shift toward oxidative metabolism [19–21]. Nevertheless, further validation of these data is required and represents an important starting point to develop new strategies to overcome chemo-resistance in cancer. Here we analyzed the functional and metabolic changes of colon cancer during the acquisition of chemoresistance to 5-FU *in vitro* and *in vivo*.

## RESULTS

### 5-FU resistance of HT29 colorectal cancer cells is associated with metabolic reprogramming toward OXPHOS

**Figure 1:**
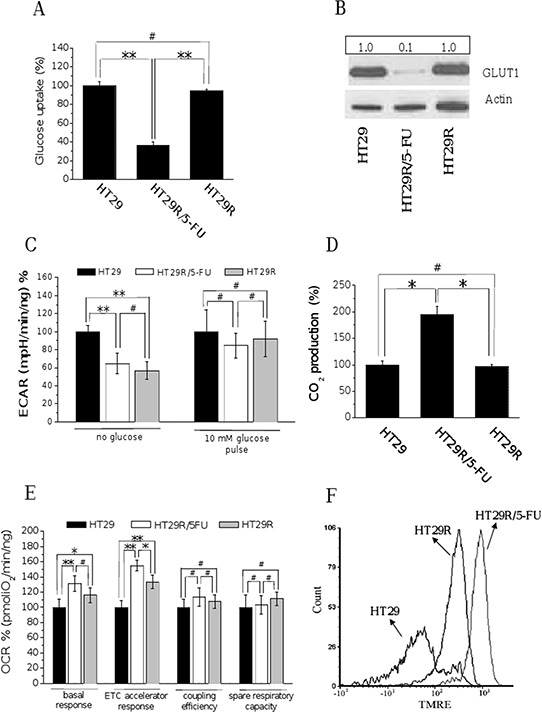
Chronic treatment with 20 μM 5-FU of HT29R decreases glucose utilization and commits cells to a respiratory metabolism For HT29, HT29R and HT29R/5-FU cell cultures has been determined: **A.** glucose uptake measured using (U-14C)deoxy-D-Glucose; the amount of radiolabeled glucose incorporated by cells was determined using a liquid scintillator analyzer. Data were obtained normalized respect to cell number and reported as percentage respect to the control experiment. **B.** glucose transporter-1 (GLUT1) expression levels determined by western blot analysis; the ratio between GLUT1 and Actin expression levels was reported in the box showed above the figure; **C.** extracellular acidification rates (ECAR) in the presence or not of glucose determined by XF analysis; **D.** ^14^CO_2_ production evaluated following incubation cancer cells with radiolabeled D-(U-14C)glucose; data were obtained normalized respect to cell number and reported as percentage respect to the control experiment **E.** Oxygen consumption rate (OCR) evaluated by XF analysis performed under mitochondrial stress conditions; **F.** mitochondrial membrane potentials evaluated through the TMRE Mitochondrial. Membrane Potential Assay Kit and a flow cytometry apparatus. Data reported in the graphics A, C, D, and E, represent the mean value +/− S.E.M. All experiments were carried out at least in triplicate. (**p < 0.01; *p < 0.05; ^#^*p* > 0.1).

To decipher the metabolic changes associated with resistance to therapy in colorectal cancers, we used a model of HT29 human colon cancer cells resistant to 5-FU (Suppl. Fig. 1A, 1B, 1C). The ability of selected cells to survive to treatment with 20 μM 5-FU was verified every week. We analyzed the metabolic profile of parental and 5-FU resistant cells before and after acute treatment with 20 μM 5-FU, excluding parental cells, which are 95% dead after 72 h (Suppl. Fig. 1B). Acute treatment of 5-FU-resistant cells was associated with a strong decrease of glucose uptake and consumption (Fig. 1A and Suppl. Fig. 2A), as well as with a decrease of GLUT-1 expression (Fig. 1B, and Suppl. Fig. 2B).

Extracellular flux analysis (Seahorse technology) showed that, under glucose deprivation, resistant cells significantly reduced lactate production, regardless of the presence of 5-FU in the growth medium. A 10 mM glucose pulse attenuated the difference in lactate production between sensitive and resistant cells: resistant cells continued to reproducibly produce less lactic acid compared to sensitive cells, but the difference was not statistically significant (Fig. 1C). Hovewer, direct quantification of released lactate reveals that 5-FU treated cells decrease production of lactate with respect to parental cells (Suppl. Fig. 2C). In addition, it is interesting to note that carbon dioxide production from (^14^C)glucose was higher in resistant cells treated with 5-FU compared to untreated and parental cells (Fig. 1D), suggesting that treatment with 5-FU increases the flux of glucose-derived carbons in the TCA cycle. Overall, these findings indicate that treated resistant cells reduce the glucose consumption and redirect pyruvate into the Krebs cycle instead of converting it into lactate.

Seahorse analysis further revealed that resistant cells have a significantly higher oxygen consumption rate, both under basal and carbonyl cyanide-4-(trifluoromethoxy)phenylhydrazone (FCCP)-uncoupled conditions (Fig. 1E and Suppl. Fig. 2D). The oxygen consumption rate (OCR) analysis under treatment with oligomycin, an ATP-synthase inhibitor, indicates an increased coupling between oxygen consumption and ATP production in resistant cells. Resistant cells maintain a spare respiratory capacity, which refers to their ability to upregulate OXPHOS for extra ATP under conditions of stress and/or increased energy demands [22; 23], similar to sensitive cells. All these characteristics suggest a higher mitochondrial activity in 5-FU resistant cells upon exposure to the drug, further confirmed by direct measurement of the mitochondrial membrane potential using the TMRE dye (Fig. 1F). In keeping, we also confirmed an increase in mitochondrial mass in resistant cells in response to 5-FU (Suppl. Fig. 3A and 3B). Based on the recent indications of the role played by the SIRT1-PGC1-α axis in mitochondrial biogenesis correlated with chemoresistance [24], we confirmed the activation of this pathway also in our chemoresistant cells (Suppl. Fig. 3C). Analysis of NAD+/NADH ratio finally confirms the increased mitochondrial activity in chemoresistant cells upon treatment with 5-FU (Suppl. Fig. 3D).

To further validate our findings in another model, we established 5-FU resistant HCT116 colon cancer cells by prolonged culture in the presence of 20 μM 5-FU. Acute treatment of 5-FU resistant cells decreased cell proliferation in the absence of cytotoxic effects. Conversely, parental HCT116 cells showed a robust apoptotic response analogous to that observed with the HT29 model (Suppl. Fig. 4A, 4B, 4C). Metabolic characterization of HCT116 parental and 5-FU resistant cells shows reduced glucose consumption in drug-resistant cells (Suppl. Fig. 4D). Moreover, the decrease of glucose consumption was associated with increased OXPHOS activity (Suppl. Fig. 4E), as well as increased mitochondrial membrane potential (Suppl. Fig. 4F). To confirm *in vivo* resistance of HT29R cells to 5-FU, a xenograft model was established in mice and animals were randomized and treated with 10 mg/kg 5-FU. Results show robust antitumor activity of 5-FU in parental HT29 cells, accompanied by a drastic reduction of proliferation and induction of apoptosis. Conversely, in HT29R-derived tumors, 5-FU treatment only minimally reduced proliferation and did not induce apoptosis, indicating that HT29R cells retain their intrinsic resistance to 5-FU-induced toxicity *in vivo* (Suppl. Fig. 5A, 5B, 5C).

### 5-FU treatment of HT29 resistant colorectal cancer cells causes oxidative stress associated with decreased pentose phosphate pathway (PPP)

**Figure 2:**
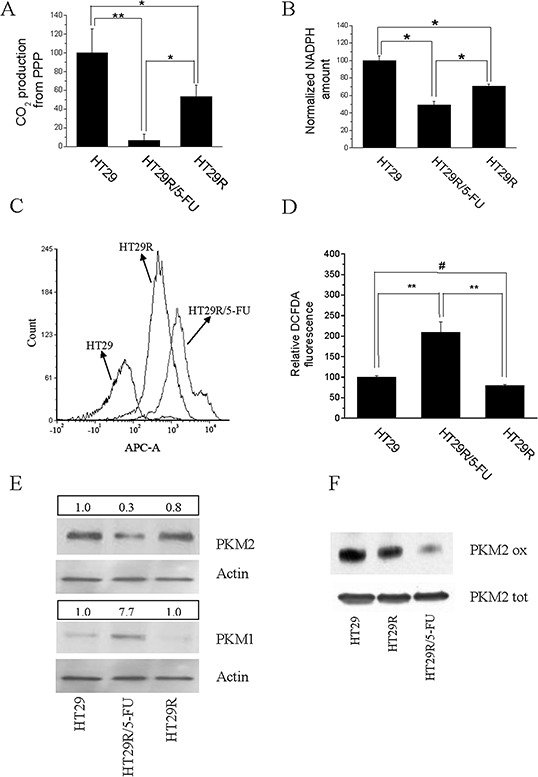
Acute treatment with 20 μM 5-FU modifies redox equilibrium of HT29R cells inhibiting PPP and NADPH synthesis For HT29, HT29R and HT29R/5-FU cell cultures has been determined: **A.** relative PPP rates, obtained measuring the differences between radiolabeled CO_2_ released after cells incubation with (6-^14^C)glucose from the CO_2_ released from catabolism of (1-^14^C)glucose. **B.** Relative NADPH levels determined through enzymatic assays. Each test was carried out in triplicate. Data reported in the figure represent the mean value +/− SEM. Data were normalized respect to protein content and reported as percent respect to the control experiment. **C.** Quantification of ROS levels carried out using DCFDA and flow cytometry, or **D.** measuring the fluorescence emitted from cells using a microplate fluorometer (Fluoroskan Ascent, ThermoFischer); **E.** expression levels of PKM1 and PKM2 evaluated by western blot; **F.** evaluation of redox status of PKM2 isoenzyme with BIAM. Data reported in the graphics A, B, and D, represent the mean value +/− S.E.M. All experiments were carried out at least in triplicate. (**p < 0.01; *p < 0.05; ^#^*p* > 0.1).

Metabolic deregulation of cancer cells towards a Warburg behavior has been associated with increased glucose uptake, followed by accumulation of glycolytic intermediates and fueling PPP. Activation of PPP allows cancer cells to obtain ribose-5P and NADPH, key molecules to synthesize nucleotides and sustain the rapid growth of cancer cells, as well as to resist oxidative stress, a common feature of cancer cells undergoing therapeutic regimens. To understand if the down regulation of glycolytic pathways observed in 5-FU treated HT29R cells would also impact glucose flux through PPP, consequently increasing intracellular ROS levels, we evaluated the activity of PPP through C1/C6 (^14^C)glucose cell loading. We observed that 5-FU treatment of HT29R cells caused a 90% reduction of PPP (Fig. 2A), accompanied by decreased NADPH accumulation and increased ROS production (Fig. 2B, 2C, 2D and Suppl. Fig. 6A). OXPHOS is the major source of ROS production in cells; thus, increased oxidative stress can be a consequence of increased mitochondrial respiratory activity, as demonstrated by cytofluorimetric analysis and co-staining with Mitotraker and Mitosox (Suppl. Fig. 6B and 6C). Finally, because pyruvate kinase M2 (PKM2) isoenzyme is a key mediator of the Warburg effect and is responsible for diverting glucose into PPP to fuel protein synthesis and cell growth, we evaluated PKM2 expression in resistant cells. Interestingly, we observed that 5-FU treatment of HT29R cells leads to decreased expression of PKM2, accompanied by increased expression of the more active isoenzyme PKM1 (Fig. 2E and Suppl. Fig. 7A, and 7B). In addition, PKM2 expressed by 5-FU-treated cells was further inhibited through cysteine oxidation (Fig. 2F), in agreement with the oxidative stress sustained by PPP inhibition.

### 5-FU treatment of HT29R cells elicits epithelial-mesenchymal transition and stemness phenotype

**Figure 3:**
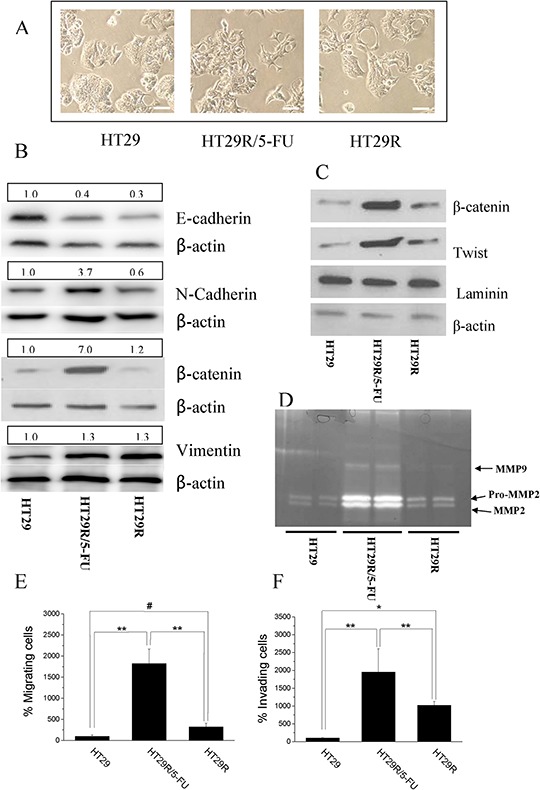
Chronic treatment with 20 μM 5-FU stimulates EMT in HT29R cells **A.** Representative images of HT29, HT29R and HT29R/5-FU cells acquired through contrast phase microscopy. Scale bar 100 μm **B.** Immunoblot of the EMT keymarkers: E-Cadherin, N-Cadherin, β-catenin, Vimentin. **C.** Analysis of nuclear localization of β-catenin and Twist performed on purified nuclear fractions. **D.** In gel-MMP-2 and MMP-9 metalloproteinases activity. Migratory **E.** and invasive **F.** abilities of either HT29, HT29R or HT29R/5-FU cells determined using Boyden chamber assay. The data reported in the figures E and F, represent the mean value +/− S.E.M. All experiments, were carried out in triplicate. (**p < 0.01; *p < 0.05; ^#^*p* > 0.1).

In order to correlate cancer cell malignancy to resistance to 5-FU treatment, we analyzed features that have been previously associated with metabolic deregulation of cells, including EMT and stemness. 5-FU treatment of HT29R cells caused a spindle-like morphology (Fig. 3A) associated with enhanced EMT markers, such as decreased E-cadherin, increased N-cadherin and vimentin (Fig. 3B and Suppl. Fig. 8A, 8B, 8C, 8D), and enhanced nuclear content of β-catenin and Twist transcription factor (Fig. 3C). In keeping with the engagement of an EMT program, 5-FU treatment of HT29R cells also caused a robust secretion of the metalloproteases (MMPs), MMP2 and MMP9 (Fig. 3D), key proteolytic enzymes driving mesenchymal motility. Consistent with secretion of MMPs, 3D motility as well as invasiveness through Matrigel barrier were both strongly activated by 5-FU treatment (Fig. 3E, 3F).

**Figure 4:**
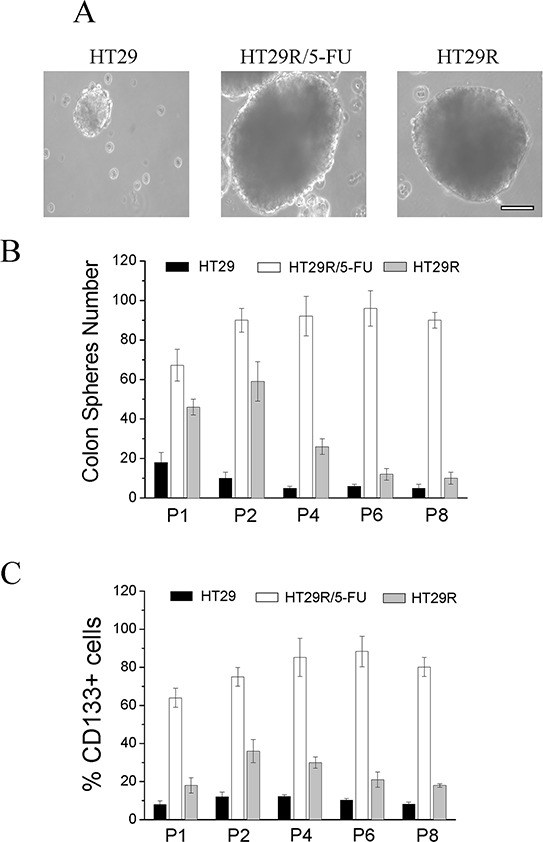
Analysis of stemness of HT29, HT29R and HT29R/5-FU cells Resistant cells were treated for 72 h with 20 μM 5-FU and then assayed for their ability to form colon-spheres. **A.** contrast phase microscopy images of sphere obtained after 21 days (P1 generation). Scale Bar 200 μm. To evaluate their self-renewal ability, single spheres obtained in P1 generation were withdrawn, mechanically disrupted and then re-plated. The evaluation of colon-spheres forming ability of cancer cells, was carried out by counting the number of colon-spheres using a contrast phase microscopy. **B.** Quantification of colon spheres number at different passages. Data reported in the figures represent the mean values +/− S.E.M from at least three independent experiments. **C.** cytofluorimetric analysis of CD133+ positive cells in colon-spheres of different passages. Data reported in the figures represent the mean values +/− S.E.M from at least three independent experiments.

EMT has been associated in several cancer models with stem-like traits, such as expression of stemness markers, self-renewal capacity, and formation of anchorage-independent spheres. We observed that cells resistant to 5-FU had greatly enhanced the ability to form anchorage-independent spheres, and that maintenance of the stem-like phenotype, as well as the expression of the CD133 marker, was associated with 5-FU treatment (Fig. 4A, 4B, 4C). Indeed, analysis of spheres across 8 passages indicated that chronic treatment of HT29R cells with 5-FU preserved the stem-like phenotype, whereas it is evident that untreated resistant cells gradually lose their ability to form colon spheres and CD133 expression (Fig. 4A, 4B, 4C). Even if the causes of this phenomenon remain to be clarified, it is likely that chemoresistant cells need the chronic administration of the drug to maintain stem-like traits that they have acquired. Taken together, these results indicate that acquisition of chemo-resistance does not correlate *per se* with a more motile and stem-like phenotype, but administration of 5-FU to resistant cells stimulates EMT and stem-like traits.

### HT29R cells exposed to 5-FU are sensitive to inhibition of OXPHOS

**Figure 5:**
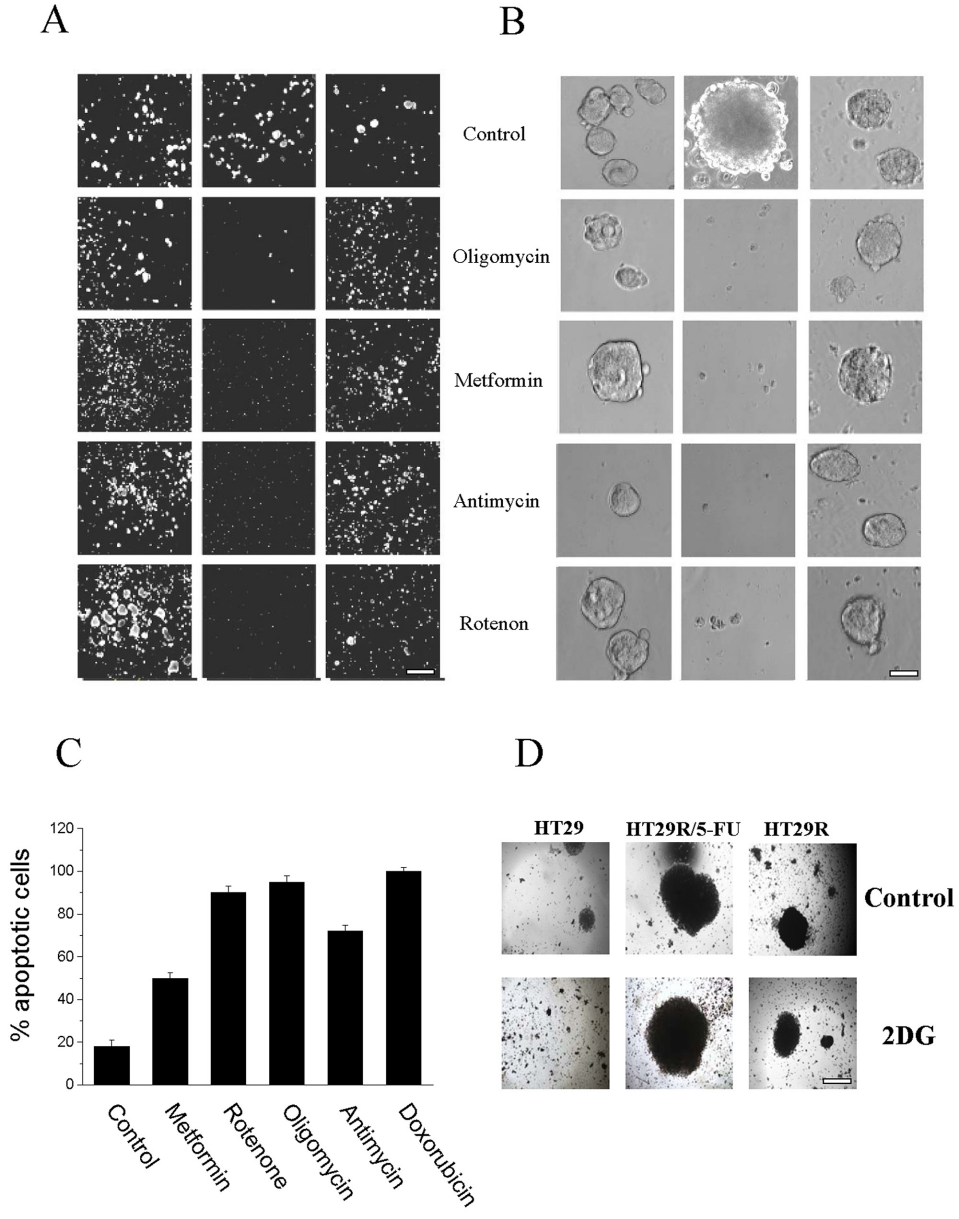
Effect of 20 μM 5-FU and OXPHOS inhibitors treatment on cells viability **A.** Calcein vital staining of cells growth in 3D; images of cells are obtained using a widefield automated microscope (ImageXpress^®^ Micro XLS System); Scale Bar 500 μm. **B.** Images of cancer cells, growth as spheres, acquired by contrast phase microscopy of HT29, treated for 3 days with or without 5 mM meftormin, 500 nM Oligomycin, 1 μM Rotenone or 1 μM Antimycin; Scale Bar 100 μm. **C.** Quantification of apoptosis relative to doxorubicin treated cells obtained using Annexin V FITC apoptosis detection kit. **D.** Images, acquired by contrast phase microscopy of cells growth as spheres, and cultured for 48 h in presence of 5 mg/ml 2 DG; Scale Bar = 200 μm.

To understand the role of metabolic reprogramming and activation of OXPHOS in 5-FU treated HT29 cells, we treated tumor cells in presence or absence of 5-FU with different OXPHOS inhibitors: Complex I inhibitors metformin and rotenone, Complex III inhibitor antimycin, or Complex V inhibitor oligomycin. Calcein staining showed that OXPHOS inhibition affected the ability of cells treated with 5-FU to grow in 3D conditions, but it did not impair clonogenity of either parental or untreated resistant cells (Fig. 5A, 5B). We also confirmed that HCT116 colon cancer cells resistant to 5-FU were addicted to respiratory metabolism (Suppl. Fig. 9A, 9B). In addition, we observed that treatment with OXPHOS inhibitors strongly affected the viability of both HT29 and HCT116 resistant cells treated with 5-FU (Fig. 5C and Suppl. Fig. 9C). Administration of 5 mg/ml 2-deoxyglucose, an inhibitor of Warburg-like behavior, for 48 h did not affect the spherogenic potential of 5-FU treated HT29R cells (Fig. 5D), thereby confirming that colon-spheres are likely addicted to OXPHOS to survive 5-FU treatment.

**Figure 6:**
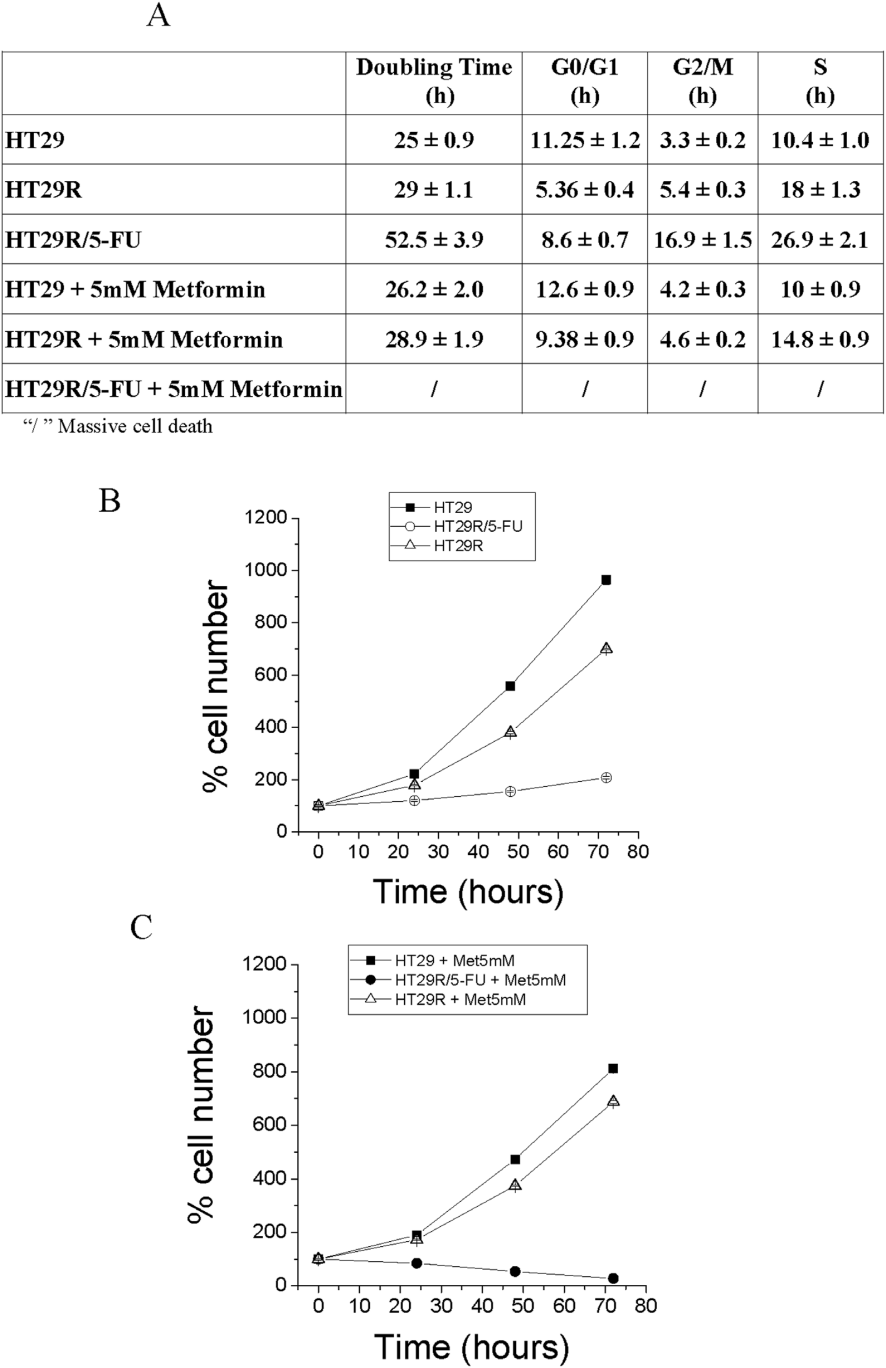
Cell proliferation and cell cycle analysis of HT29, HT29R and HT29R/5-FU treated with metformin **A.** Cell cycle analysis. After 72 hours, parental, resistant and resistant cells treated with 5-FU were fixed with ethanol and then stained with propidium iodide. Analyses of samples are carried out using a flow cytometry apparatus (FACSscan, BD Biosciences). Cell distribution between cell cycle phases was carried out using ModFit LT™ Highlights program. All test were carried out in triplicate. Data reported in the Table represent the mean values +/− S.E.M. Duplication time, was determined as described in Materials and Methods section. **B.** and **C.** Evaluation of HT29, HT29R and HT29R/5-FU cells growth rate in the absence (B) or presence (C) of 5 mM metformin. Number of cells is determined by cell counting using a contrast phase microscope. Values reported represent the mean value +/− S.E.M. All tests were carried out in triplicate.

To investigate the effect of combination treatment with 5-FU and metformin on cell growth and survival, we analyzed the effect of metformin on cell cycle and cell proliferation. The results indicate that 5-FU induces an elongation of cell cycle, which correlated with decreased proliferation of HT29R cells (Fig. 6A, 6B). Combined treatment with metformin and 5-FU induced death in the HT29R cells thereby confirming the complete addiction of these cells to OXPHOS. The requirement of OXPHOS for HT29R cells was also confirmed by treatment with electron transfer chain/OXPHOS inhibitors, as rotenone, antimycin A and oligomycin (Suppl. Fig. 10A, 10B, 10C, and 10D).

**Figure 7:**
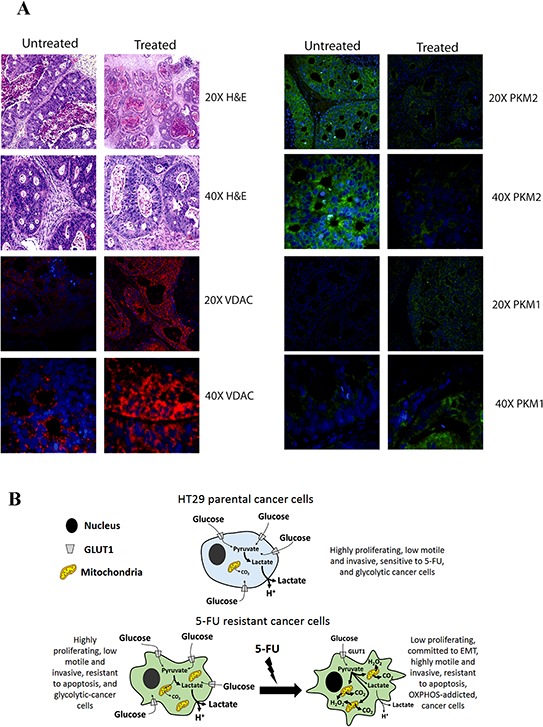
**A.** Analysis of PKM2, PKM1 and VDAC expression levels in samples derived from xenopatients. Representative H&E and immunofluorescence of patient derived xenografts untreated or treated with 5-FU. Images of samples were acquired using a fluorescence microscopy Nikon Eclipse. **B.** Scheme of metabolic profile of parental and resistant cells. The pictures highlight the metabolic profile of HT29 parental cells as well as the conversion of resistant cells from a glycolytic toward respiratory metabolism upon chronic treatment with 5-FU.

To validate our observations *in vivo*, we analyzed samples obtained from colon cancer xenotransplants using chemo-naïve stage III human colon adenocarcinoma treated weekly with 5-FU (50 mg/kg) and oxaliplatin (5 mg/kg) for their mitochondrial content and for PKM1/2 expression. 5-FU-treated tumors expressed high levels of PKM1 and low levels of PKM2 compared to untreated tumors. In addition, 5-FU-treated tumors showed high levels of VDAC1 expression, a well-established mitochondrial protein marker, confirming that treatment with 5-FU increased mitochondrial content in resistant cells *in vivo* (Fig. 7).

## DISCUSSION

Data reported herein indicate that under chronic treatment with 5-FU, *i)* colon cancer cells resistant to 5-FU plastically shift their metabolism towards OXPHOS; *ii)* revert their addiction to a Warburg-like metabolism by inverting the ratio between PKM2 and PKM1 glycolytic enzymes; *iii)* this metabolic adaptation is related to the commitment of cancer cells to EMT, increased motility and achievement of stem-like traits; *iv)* OXPHOS inhibition, in combination with 5-FU, dramatically affects survival of resistant cells. All these effects are not observed or dramatically attenuated in untreated resistant cells.

Expression of PKM2 in tumor and proliferating cells sustains Warburg metabolism. The inversion of the ratio between PKM1 and PKM2 in favor to the latter, as well as tyrosine phosphorylation or cysteine oxidation of PKM2, have been correlated with high cellular proliferation. Our data indicate that in resistant cells, the continued administration of 5-FU leads to increased expression of PKM1 and decreased expression of PKM2. Moreover, PKM2 activity is also inhibited by cysteine oxidation.

Consistent with the reversal of the PKM2/PKM1 ratio, 5-FU-treatment resistant cells showed a higher respiratory capacity compared to parental cells but also a surprising metabolic plasticity. Indeed, the shift towards OXPHOS was strongly attenuated upon removal of the drug, rescuing a Warburg-like phenotype. In keeping with the advantage given by Warburg metabolism in terms of accumulation of glycolytic intermediates, the proliferation rate of resistant cells is similar to that of parental cells in the absence of 5-FU, and these cells rely on glycolytic pathway to satisfy their metabolic needs. Conversely, 5-FU-treated resistant cells strongly decrease their proliferation rate and shift toward OXPHOS. Data obtained from patient-derived xenograft mouse models confirmed that treatment with 5-FU correlated with decreased PKM2 and increased PKM1 expression, as well as with increased mitochondrial mass (VDAC1), thus validating data obtained *in vitro*.

Notably, 5-FU-treated resistant cells show elevated levels of ROS in response to drug administration and a decrease in NADPH levels, consistent with the robust decrease in PPP in 5-FU treated resistant cells. This finding is in apparent contradiction with the metabolic behavior of these cells. Indeed, Warburg metabolism has been correlated with accumulation of glycolytic intermediates due to the expression of the rate-limiting PKM2, thereby shunting glucose into PPP to obtain pentoses for nucleic acids synthesis, anabolism and proliferation. In addition, up-regulation of PPP is a consolidated mechanism exploited by several cancer cells to control intracellular ROS levels, thereby protecting themselves from oxidative stress and death [25]. We argue that in 5-FU-treated resistant cells the reduction of glycolytic flux is the main inhibitor of PPP, which results in the observed decrease in NADPH synthesis and increase of ROS that are mainly produced by leaking electrons from the electron transport chain. Nevertheless, we cannot exclude that the limited NADPH availability could *per se* prevent the regeneration of antioxidants, thereby contributing to elevate ROS levels in 5-FU resistant treated cells. We explain these findings with the low proliferation rate of 5-FU treated resistant cells, which likely do not need to fuel anabolic pathways starting from PPP (nucleic acid synthesis) or from glycolytic (aminoacids) intermediates, at least as highly proliferating cells. This metabolic adaptation allows OXPHOS-addicted cancer cells to easily survive drug treatments, but leaves cells susceptible to inhibitors of OXPHOS.

In the last decades, several studies demonstrated that deregulation or mutations of several metabolism-related enzymes are common features of cancer cells, with the consequence that most of these rely on aerobic glycolysis to satisfy their metabolic needs. These features renew hope that targeting cancer cell metabolism may improve patient survival [26–28]. As tumors are heterogeneous systems in which highly proliferative cells coexist with subpopulations of quiescent or slowly proliferating cells, i. e. cancer stem cells or tumor-initiating cells, the disparate metabolic phenotypes of tumor subpopulations have attracted the interests of molecular oncologists. CCCs show a puzzled metabolic behavior, mainly due to their unusual plasticity [29; 30]. Some preliminary indications suggest that these cells, endowed with self-renewal and stem-like traits, exploit mitochondrial respiration to survive and support their low rate of proliferation, although this indication is usually indirect [31]. Indeed, several papers address the efficacy of metformin in eradicating cancer stem cells [32–34]. Metformin is an approved antidiabetic drug that inhibits mitochondrial respiration by blocking complex I of the electron transport chain. Compelling evidence demonstrates that metformin shows a preferential cytotoxic activity toward cancer stem cells, thus preventing tumor relapse [21]. The general interest of oncologists around metformin is justified by its success in recent clinical trials in some aggressive cancers [35–38], as well as by recent indications of its synthetic lethality in association with chemotherapy drugs [39–44]. We report here that metformin is active in *de novo* sensitizing resistant cells to 5-FU treatment. We argue that metformin mainly targets OXPHOS-addicted cells, as the effects of metformin treatment were phenocopied by others OXPHOS inhibitors. Indeed, all these molecules fully abrogated survival to 5-FU-resistant cells, as well as motility and achievement stem-like traits, strongly supporting the synthetic lethality of OXPHOS-inhibitors for 5-FU treatment-resistant cells.

Although we lack a mechanistic explanation of the shift towards OXPHOS of 5-FU-treated resistant colon cancer cells, we hypothesize that it is the result of adaptive metabolic reprogramming. Recent data indicate that 5-FU leads to activation of ataxia-telangiectasia mutated serine/threonine protein kinase (ATM), AMP kinase (AMPK) and PGC-1α, which could promote mitochondrial biogenesis and stimulate OXPHOS [45–47]. Our results indicate that metformin, which has been reported to inhibit complex I in OXPHOS, is almost completely ineffective against colon cancer cells in the absence of 5-FU. 5-FU administration is mandatory to achieve the metabolic reprogramming of cancer cells to OXPHOS, thereby increasing their sensitivity to metformin. This observation indicates that OXPHOS inhibition is the primary mechanism of action of metformin resulting in anti-tumor effects, while targeting AMPK plays a marginal role. Moreover, the metabolic shift towards OXPHOS is completely reversible once 5-FU is removed, confirming that metformin loses its effect when used alone.

Our observation that drug sensitivity and achievement of stem-like features correlate with a reliance on OXPHOS in colon cancer cells is in line with very recent work indicating OXPHOS as mandatory for cancer malignancy. First, oncogene ablation in a model of pancreatic cancer resulted in rapid tumor regression but favored the selection of a subpopulation of cancer cells with stem-like properties that relies on OXPHOS for survival and were responsible for tumor relapse [18]. Second, it has been reported that that expression of PGC-1α, a transcriptional coactivator that stimulate mitochondrial biogenesis and OXPHOS, increases cancer cell invasiveness and correlates with survival of circulating cancer cells, as well as their metastatic dissemination, suggesting that mitochondrial activity contributes to the development of resistant and malignant cancer phenotypes [48]. SIRT1/PGC1-α axis has also been correlated with chemoresistance and metabolic shift towards OXPHOS in liver metastasis of colon cancer [24]. Moreover, Porporato recently associated high mitochondrial activity of melanoma cells with a hypermetastatic phenotype [49]. Last, glioblastoma cells resistant to temozolomide showed a prevalent respiratory metabolism [16]. Altogether, these findings suggest that new therapeutic strategies that incorporate targeting of OXPHOS may selectively kill CCCs and yield more durable responses.

## MATERIALS AND METHODS

### Materials, cell lines and assays

HT29 colon carcinoma cells were kindly provided by M. Landriscina (University of Foggia) and authenticated by PCR/short tandem repeat analysis. HT29 resistant to 5-FU (HT29R) were selected as previously reported [50]. To ensure maintenance of resistance to 5-FU, HT29R cells were steadily grown in the presence of with 20 μM 5-FU. HCT116 were purchased from American Collection of Cell Cultures (ATCC). For 3D tissue culture, cells were maintained in low-attachment plates in media composed of 2% methylcellulose in Mammary Epithelial Basal Medium (Lonza) supplemented with 5 mM Glutamine, 10 μg/ml EGF, 10 μg/ml FGF, and 1X B27 Serum Free Supplement.

Unless specified, all reagents were from Sigma and all the antibodies were from Santa Cruz Biotechnology, except for the following: anti-PKM1, anti-PKM2 (Cell Signaling Technology), anti-CD133 (Miltenyi), anti β-catenin (BD Biosciences). Intracellular production of ROS was assayed by Cell ROX (Life Technologies) or H_2_DCF-DA as previously described [51]. Immunoprecipitation and immunoblots were performed as previously described [52].

Glucose concentration in the medium was measured using a Glucose Assay Kit (Sigma-Aldrich) and NADPH content using an NADP/NADPH Assay Kit (AbCam).

### Statistical analysis of data

Analysis of proteins expression levels, the MTT assays for determination of the IC50 values against 5-FU, tests for quantification of CO_2_, ROS, enzymatic assays for determination of cellular NADPH and NADH levels, the assays of glucose uptake, the colon sphere forming ability assays, the tests for the quantification of CD133+ expression, the assays for evaluation of cellular growth rate, the assays for the quantification of percentage of apoptotic cells, as well as the *in vivo* experiments were carried out at least in triplicate. Results are expressed as means ± S.E. M. and were analysed using the Student's *t* test. A *p* value < 0.05 was considered statistically significant.

### Apoptosis evaluation

Apoptosis was determined using Annexin V and Dead Cells Assay from Merck Millipore according to manufacturer's instructions. Cells were analyzed by flow cytometry. To determine viability of cells grown in 3D conditions, cells embedded in methylcellulose-based semisolid media were incubated for 30 minutes with 1 μM calcein (Life Technology) and quantified through ImageX press velos (Molecular Devices) apparatus.

### Colony formation assay

For colony formation assays, 500 cells were seeded into a six-well plate, treated for 3 days with 5-FU 20 μM, Oligomycin 500 nM, Metformin 5 mM, Rotenone 1 μM, or Antimycin 1 μM. The medium was replaced and cells were cultured for 9–11 days. Subsequently, cells were fixed and stained with a solution containing 1% crystal violet (Sigma–Aldrich), and 10% methanol. Colonies were photographed and counted using a GelDoc imaging system (Bio-Rad Laboratories).

### Cell migration in Boyden Chamber

Cell migration was performed with 5 × 10^4^ HT29 and HT29R/5-FU cells on 8-μm-pore Transwells (Corning) for 24 h, as previously described [51]. Chemotaxis was evaluated by counting the cells migrated to the lower surface of the filters (six randomly chosen fields). To evaluate cell invasiveness, Transwells were coated with 50 μg/cm2 of reconstituted Matrigel.

### Flow cytometry analysis

Cell cycle analysis was carried out using cytofluorimetric method. Briefly, HT29 and resistant cells were grown for 72 hours and then cells were fixed in 70% cold ethanol. After cells were re-suspended in a buffer containing 0.05 mg/ml propidium iodide, 5 μg/ml RNAase A, 0.2% v/v Nonidet P-40, 0.1% sodium citrate. Samples were analyzed by FACSscan flow cytometer apparatus (BD Biosciences). Doubling time was extrapolated using the following formula: T*d* = 24*lg2/(lg(n°cell*t1)-(n°cell*t0)). To evaluate mitochondrial membrane potential, cells were stained with 500 nM Tetramethylrhodamine, ethyl ester (TMRE) dye for 20 min at 37°C and flow cytometry was performed using a FACSscan (BD Biosciences). To evaluate the fraction of CD133 positive cells, 1 × 106 HT29, HT29R and HT29R/5-FU cancer cells were labelled with FITC- anti-CD133 (human clone: AC133) antibodies for 1 h at 4°C in the dark. Then, cells were washed and analysed by flow cytometry using a FACSscan (BD Biosciences).

### Glucose uptake

One hundred thousands HT29 or HT29/5-FU cells were cultured for 72 h. Cells were incubated for 15 min at 37°C in a buffer solution (140 mmol/L NaCl, 20 mmol/L Hepes/Na, 2.5 mmol/L MgSO_4_, 1 mmol/L CaCl2, and 5 mmol/L KCl, pH7.4) containing 0.5 μCi/mL (U-^14^C)deoxy-D-Glucose (PerkinElmer). Cells were lysed in 0.1 mol/L NaOH and assayed to determine the amount of (U-^14^C)deoxy-D-Glucose incorporated using a liquid scintillation analyzer (Tri-Carb 2800TR, PerkinElmer).

### Detection of released CO_2_ by radioactive glucose

One hundred thousands HT29 cells treated or not with 5-FU were cultured for 72 h and then 0.2 μCi/mL D-(U-^14^C)glucose was added for 15 min at 37°C. Each plate had a taped piece of Whatman paper facing the inside of the dish wetted with 100 μL of phenyl-ethylamine-methanol (1:1) to trap CO_2_. Then, 200 μL of 4M H_2_SO_4_ was added to cells and incubated at 37°C for 1 h to permit release of ^14^CO_2_. Finally, Whatman paper was removed and transferred to scintillation vials for counting. The amount of ^14^CO_2_ trapped on the paper dishes was determined using a liquid scintillator analyzer (Tri-Carb 2800TR, PerkinElmer)

### Extracellular Flux (XF) analysis (Seahorse technology)

The Oxygen Consumption Rate (OCR, pmolesO_2_ consumed/min) and the ExtraCellular Acidification Rate (ECAR, mpH/min) were determined by using the XF24 Extracellular Flux Analyzer (Seahorse Bioscience) according to manufacture's instructions. Cells were plated at 5 × 10^4^ cells/well on XF24-well microplates in standard medium. After one day incubation, before were washed three times with an unbuffered assay medium (p*H* = 7.4) and conditioned for 1 h at 37°C without CO_2_ and assayed. After XF measurements, protein content from each well was determined by using Bradford assay.

### PPP analysis

One hundred thousands HT29 cells treated or not with 5-FU were cultured for 72 h, serum starved and incubated with 0.5 μCi/ml D-(1-^14^C(U))glucose and 0.5 μCi/ml D-(6-^14^C(U))glucose (PerkinElmer) for 1 h at 37°C. The extent of PPP metabolic flux was obtained by subtracting the CO_2_ developed from (6-^14^C)glucose from the CO_2_ released from (1-^14^C)glucose.

### MMP zymography

Culture media was loaded on 8% SDS-PAGE gels copolymerized with 0.1% (w/v) type A gelatin. Gels were incubated in 50 mmol/L Tris-HCl (pH 7.4), 200 mmol/L NaCl, and 5 mmol/L CaCl2 at 37°C for 24 hours, stained with 0.1% Comassie blue in acetic acid, methanol and distilled water (1:2:3). Destained gels were scanned with Quantity-One Image Analysis software (Bio-Rad).

### Detection of PKM2 oxidation by carboxymethylation

One million HT29 cells were lysed for 15 min with oxygen-free lysis buffer containing 100 μM N-(biotinoyl)-N0-(iodoacetyl) ethylenediamine (BIAM; Molecular Probes). After clarification, PKM2 was immunoprecipitated from 500 μg of total protein and redox state was revealed by HRP-conjugated streptavidin (Pierce).

### Immunofluorescence analysis

Formalin-fixed tumors were dehydrated and paraffin embedded according to standard procedures. 5-μm slices were cut using a microtome, rehydrated, and subjected to antigen unmasking by heating at 95°C for 30 minutes with a commercially available antigen unmasking solution (Citra). Samples were incubated with primary antibodies, washed, and incubated with fluorescent labelled secondary antibodies. Nuclei were stained through 4′,6-diamidin-2-fenilindolo (DAPI) incubation and slices were mounted. Immages of samples were acquired using a fluorescence microscopy Nikon Eclipse Ni, equipped with a digital camera (Hamamatsu Digital Camera C11440).

### Colon sphere assay

One thousand HT29 cells were plated in stem cell medium (SCM) MEBM (Lonza) supplemented with 2 mM glutamine (Invitrogen), B27 (Invitrogen), 20 ng/ml hEGF (PeproTech), 20 ng/ml hFGF (PeproTech), 5 μg/ml Insulin (Roche), 0.5 μM hydrocortisone (Sigma), 100 μM β-mercaptoethanol (Sigma), 4 μg/ml heparin (Sigma). Methocult M3134 (StemCell Technologies) was added to SCM (final concentration 0.8%) to keep tumour cells growing as clonal spheres. After 7 days cultured tumour spheres were collected, digested with 0.05% trypsin (Gibco) to single cells and re-plated in culture.

### Establishment of xenografts from human tumors

Excess tumor tissues not needed for clinical diagnosis from colorectal cancer resection specimens were cut into 2- to 3-mm^3^ pieces in antibiotic-containing Roswell Park Memorial Institute (RPMI) medium. Pieces of non-necrotic tissue were selected and immersed in Matrigel. Under anesthesia with isofluorane, tumors were implanted into 5- to 6-week-old female Nu+/Nu+ mice through a small incision creating a subcutaneous pocket in each side of the lower back (one tumor fragment per pocket). Tumors were harvested and stored for biological assays when they reached 1,500 mm^3^ in diameter. This set of xenografts were called F1. Additional xenograft generations were obtained for subsequent expansion and drug treatment. The research protocol was approved by the MD Anderson Cancer Center Institutional Review Board and informed consent was collected from all patients enrolled in the patient xenograft study protocol. The research protocol was also approved by the MDACC Animal Use and Care Committee and animals were maintained in accordance to guidelines of the American Association of Laboratory Animal Care. Xenografts from subsequent mouse-to-mouse passage were allowed to grow to a size of 200 (or 150–300) mm^3^. 5-FU (50 mg/kg) was administered as a weekly intraperitoneal injection. Mice were sacrificed after 18 days of treatment.

## SUPPLEMENTARY FIGURES


