# Supplementary material for: 5-Fluorouracil resistant colon cancer cells are addicted to OXPHOS to survive and enhance stem-like traits
Source: Oncotarget. 2015 Oct 21;6(39):41706–21. doi: 10.18632/oncotarget.5991 (PMC4747183; doi:10.18632/oncotarget.5991)
Supplement: Supplementary file 1 [file oncotarget-06-41706-s001.pdf]

SUPPLEMENTARY FIGURES

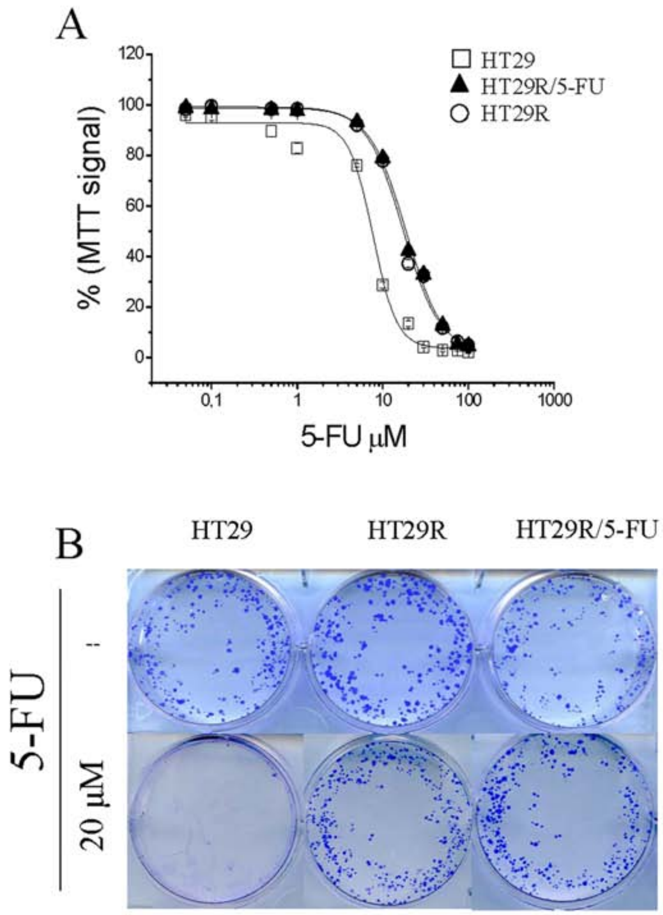

C

Cells	Apoptosis (%)
**HT29**	**5.0 ± 0.1**
**HT29R**	**10 ± 1.0**
**HT29/5-FU**	**86 ± 3.2**
**HT29R/5-FU**	**8.0 ± 0.7**

Supplementary Figure S1: To deeply investigate the difference of drug response between HT29 and HT29R cells, different analyses were conducted. A. The 5-FU IC_{50} values were determined on HT29, HT29R and HT29R cultured in the continuous presence of 20 µM 5-FU. Results evidenced lack of difference in terms of IC_{50} between resistant cells growth with or without 5-FU (IC_{50} = 18.8 ± 1.0 µM and 17.3 ± 1.2 µM, respectively) and both cases the IC_{50} was higher than that of parental cells (IC_{50} =7.7 ± 0.7 µM). **B.** To assess the effect of 5-FU on cell clonogenicity, HT29 and HT29R cells were seeded at low density and treated with 20 µM 5-FU. Results evidenced that 5-FU robustly reduced the number of colonies only on parental cells. **C.** In order to understand if the reduction of the number of colony is related to a reduction of proliferation rate or to an increase of apoptosis, we conducted a cytofluorimetric quantification of apoptotic parental and resistant cells untreated or treated with 20 µM 5-FU. Apoptosis was evaluated using Annexin V and Dead Cells Assay from Merck Millipore according to manufacturer's instructions. Cells were analyzed by flow cytometry, using a FACS Canto flow cytometer (BD Biosciences). The results revealed that 5-FU induced massive apoptosis in parental HT29 cells only. Data reported in the table represent the mean value ± S.E.M. All experiments were carried out in triplicate.

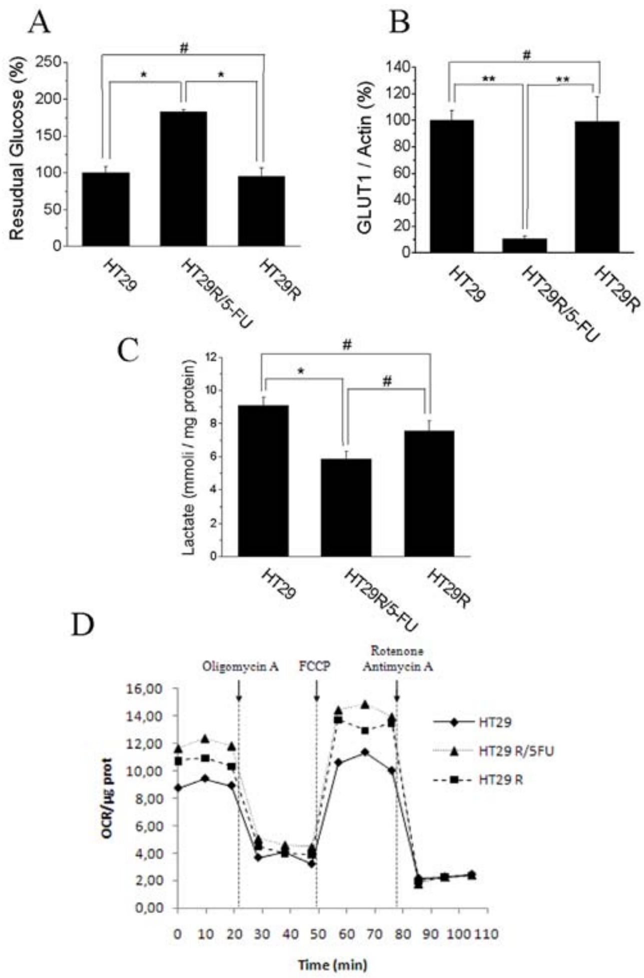

Supplementary Figure S2: A. Determination of glucose concentration in the growth medium. To evaluate the glucose consumption, 8 × 10^4 cells were plated on P35 dishes and grown at 37°C. After 72 hours, an aliquot of growth medium was withdrawn from each samples and analyzed for glucose content using the Glucose Assay Kit (Sigma-Aldrich). Data were normalized with respect to cell number. All test were carried out in triplicate. Data reported in the figure represent the mean value +/− S.E.M. (*$p < 0.05$; $^{\#}p > 0.1$). **B.** Quantification of GLUT1 expression levels. Parental and resistant cells were plated in P35 cell culture dishes and growth for 72 hours; resistant cells were growth either in the absence or in the presence of 20 µM 5-FU. After 72 hours, all cells dishes were washed with cold PBS and lysated using 1× Sample Buffer. Proteins were separated by SDS-PAGE electrophoresis and then transferred on PVDF membrane through Western Blot. Membrane were probed using specific antibodies able to bind specifically GLUT1 glucose transporter (sc7903, Santa Cruz). Detection of protein was carried out using Clarity ECL Western Blot assay kit (Biorad). Quantification of western was carried out using Kodak MI software program. Data reported represent the mean values +/− S.E.M. All experiments were performed in triplicate. (**$p < 0.01$; $^{\#}p > 0.1$). **C.** Quantification of lactate released in the growth medium. HT29, HT29R/5-FU and HT29R cells were plated on P35 culture dishes (8 × 10^4 cells for dishes). After 72 hours, aliquot of growth medium was withdrawn to determination of lactate released. Quantification was carried out using YSI 2950 Analyzer (YSI Life Sciences). Also in this case, data reported represent the mean values +/− S.E. M. determined from data obtained from three independent experiments (*$p < 0.05$; $^{\#}p > 0.1$). **D.** Extracellular flux (XF) analysis performed under mitochondrial stress condition. 1 day before XF analysis, cells were seeded at 5 × 10^4 cells/well on 24-well Seahorse plate with 200 µl/well of standard culture medium, containing 20 µM 5-fluorouracil only for HT29 R/5FU. 1 h before XF analysis cells were washed three times and incubated with the XF unbuffered assay medium without pyruvate and sodium bicarbonate at 37°C without CO_2. XF analysis was performed according to Mitostress test protocol (Seahorse Bioscience) consisting in three OCR measurements in basal condition followed by three OCR measurements after each injection of 2 µM oligomycin A, ATP synthase inhibitor, 0.5 µM FCCP, uncoupler ionophore, and a mixture of 1 µM Rotenone and 1 µM Antimycin A, ETC inhibitors. OCR (pmolesO_2/min). After XF measurements, medium from each well was removed and 70 µl lysis buffer/well (20 mM Tris, 1% Triton X-100, protease inhibitor cocktail, pH 7.4) were added. Protein concentration was analysed by using Bradford assay, in order to normalize OCR and ECAR to protein content. Parameters were calculated as average from at least three independent experiments, each performed in seven replicates per cell type/condition. The plot shows a representative profile from at least three independent experiments.

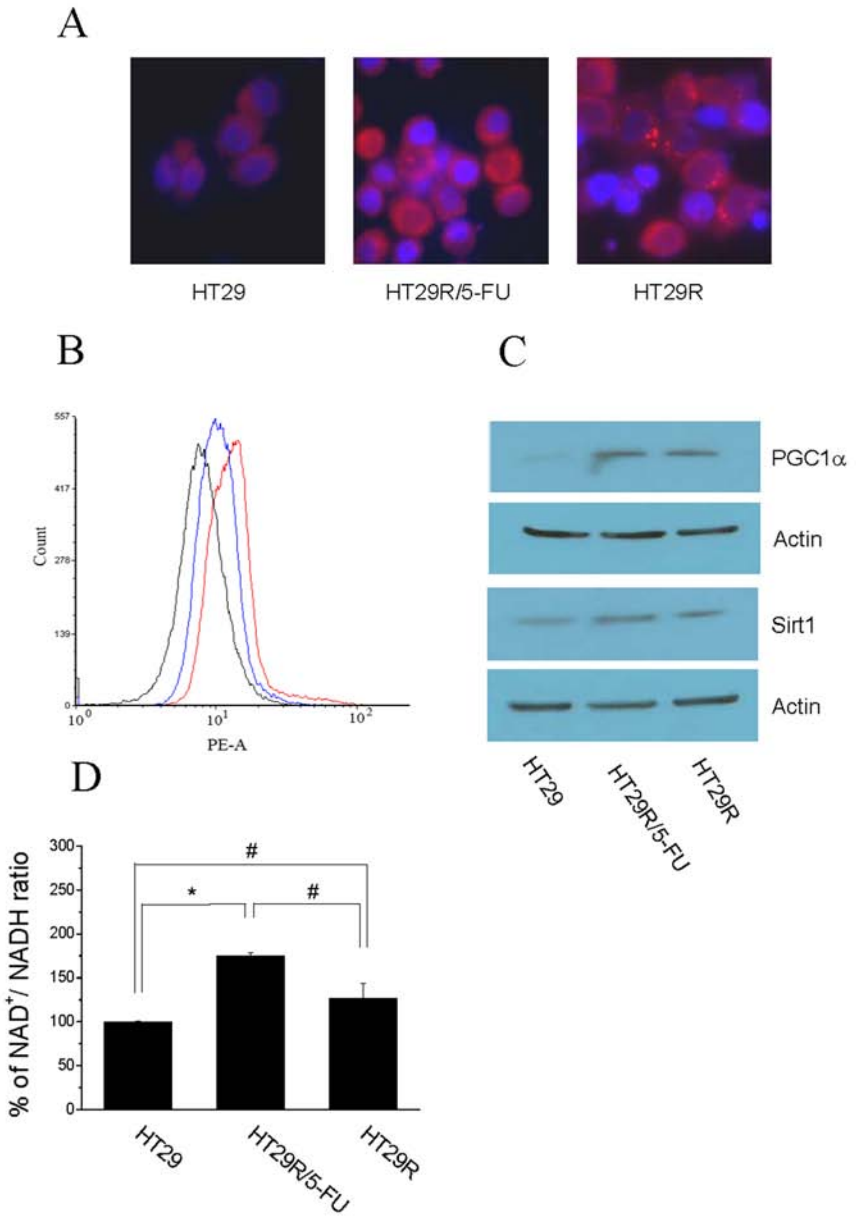

Supplementary Figure S3: A. Detection of cancer cells mitochondrial number. HT29, HT29R/5-FU and HT29R cells (1×10^5) were plated on coverslips inside a Petri dish filled with culture medium. After 72 hours, growth medium was removed, replaced with medium containing MitoTrackerR probe (500 nM, final concentration) and incubate for 20 minutes under growth conditions. Then, samples were washed with PBS and analysed by fluorescence microscope Nikon Eclipse Ni, equipped with a digital camera (Hamamatsu Digital Camera C11440). Scale bar 20 µm. **B.** Quantitative analysis of cancer cells mitochondrial content, 1 x10^5 cells were grown for 72 hours and then incubated for 30 minutes with 500 nM of MitoTrackerR. The mitochondrial mass was evaluated by flow cytometry using BD LSRFortessa (BD Bioscience). Data reported are representative images of experiments performed in triplicate. **C.** Quantification of SIRT1, PGC1αand PPARα expression levels. Cells were grown in the same condition as above. After 72 hours, cells dishes were washed with cold PBS and lysed using 1× SDS Laemli sample buffer. Proteins were separated by SDS-PAGE electrophoresis and then transferred on PVDF membrane through Western Blot. Membrane was probed using specific antibodies against SIRT1, PGC1α. **D.** Determination of NAD+/NADH ratio. After 72 hours of growth, cells were washed with cold PBS and lysed. Assay of NAD+ and NADH levels was carried out using the NAD^+/NADH Quantification Kit (Sigma Aldrich). Each experiment was performed in triplicate. Data reported in the graphic represent the mean value ± S.E.M. Data were reported as percentage of control. ($^*p < 0.05$; $^\#p > 0.1$).

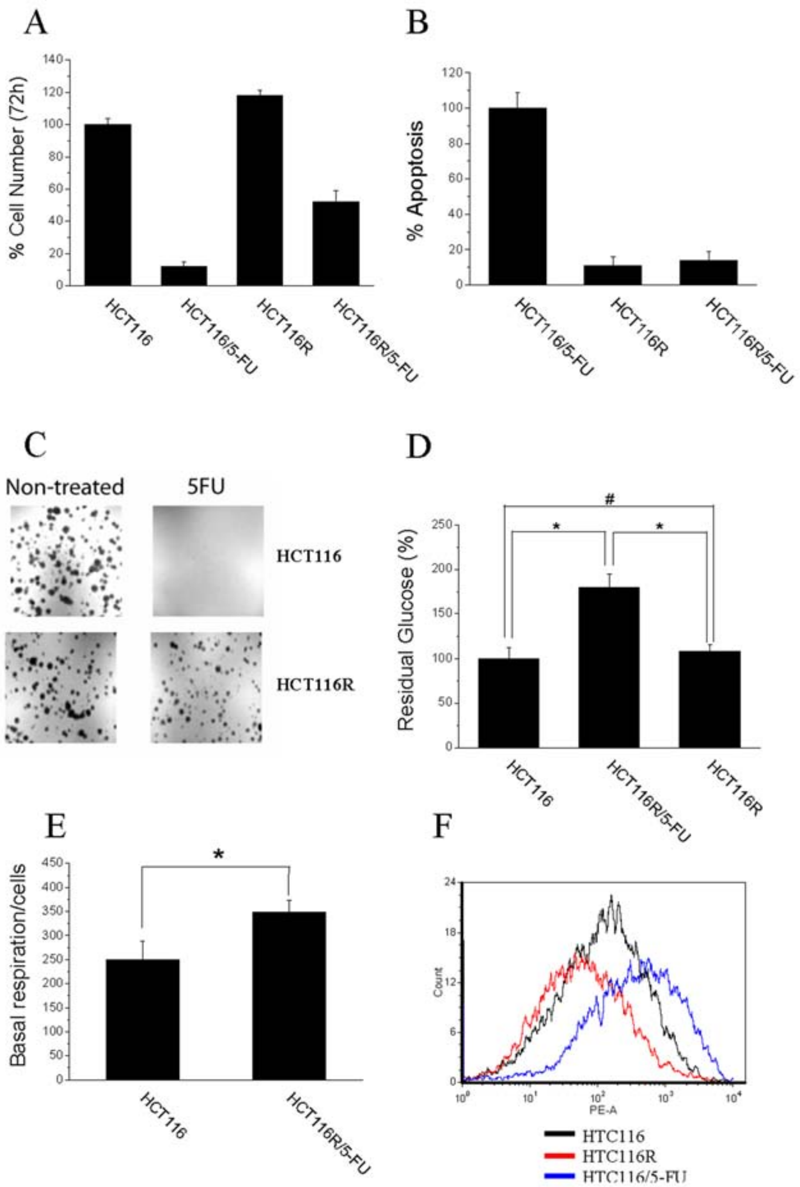

Supplementary Figure S4: Characterization of HCT116 5-FU resistant cells. To further validate our data we generated a model of HCT116 5-FU-resistant colon cancer cells selected by prolonged culture in the presence of 20 µM 5-FU. **A.** Evaluation of cell number after 72 hours of growth. Parental and HCT116R cells were grown for 72 hours in the absence or in the presence of 20 µM 5-FU before cell counting. For each experiment, cells number was normalized with respect to control experiment. Data reported represent the mean value +/− S.E.M. ($n = 3$). **B.** Determination of apoptotic cell number. Experimental condition were the same as above. After 72 hours of growth, the number of apoptotic cells weas determined tby flow cytometry using the Dead Cells Assay from Merck Millipore. Data obtained were normalized with respect to control experiment (HCT116 parental cells treated with 20 µM 5-FU). Data reported represent the mean value +/− S.E.M. ($n = 3$). 72 h acute treatment with 20 µM 5-FU cells causes a decrease of cell proliferation in both cell lines; nevertheless, while parental HCT116 cells show a robust apoptotic response, analogous to what we observed in the HT29 model, viability of HCT116 resistant cells was not significantly affected by drug treatment. **C.** Colony assay tests. Cells have been seeded as reported in material and methods, allowed to grow for 72 h and stained with crystal violet before taking pictures. **D.** Quantification of glucose consumption after 72 h of growth. The quantification of glucose in the medium of HCT116 parental and 5-FU resistant treated and untreated cells, shows reduced glucose consumption in resistant cells compared with parental cells ($*p < 0.05$; $^{\#}p > 0.1$). **E.** Seahorse analysis of respiratory ability (OCR) of parental and resistant cells treated with 5-FU. Analysis was carried out as in Suppl. Figure S(2D). Similarly to HT29 cells, also HCT116 resistant cells increase basal respiration upon treatment with 5-FU. **F.** Analysis of mitochondrial membrane potential carried out using the mitochondrial membrane potential-sensitive TMRE probe as reported in material and methods. Quantitative analyses were carried out by flow cytometry.

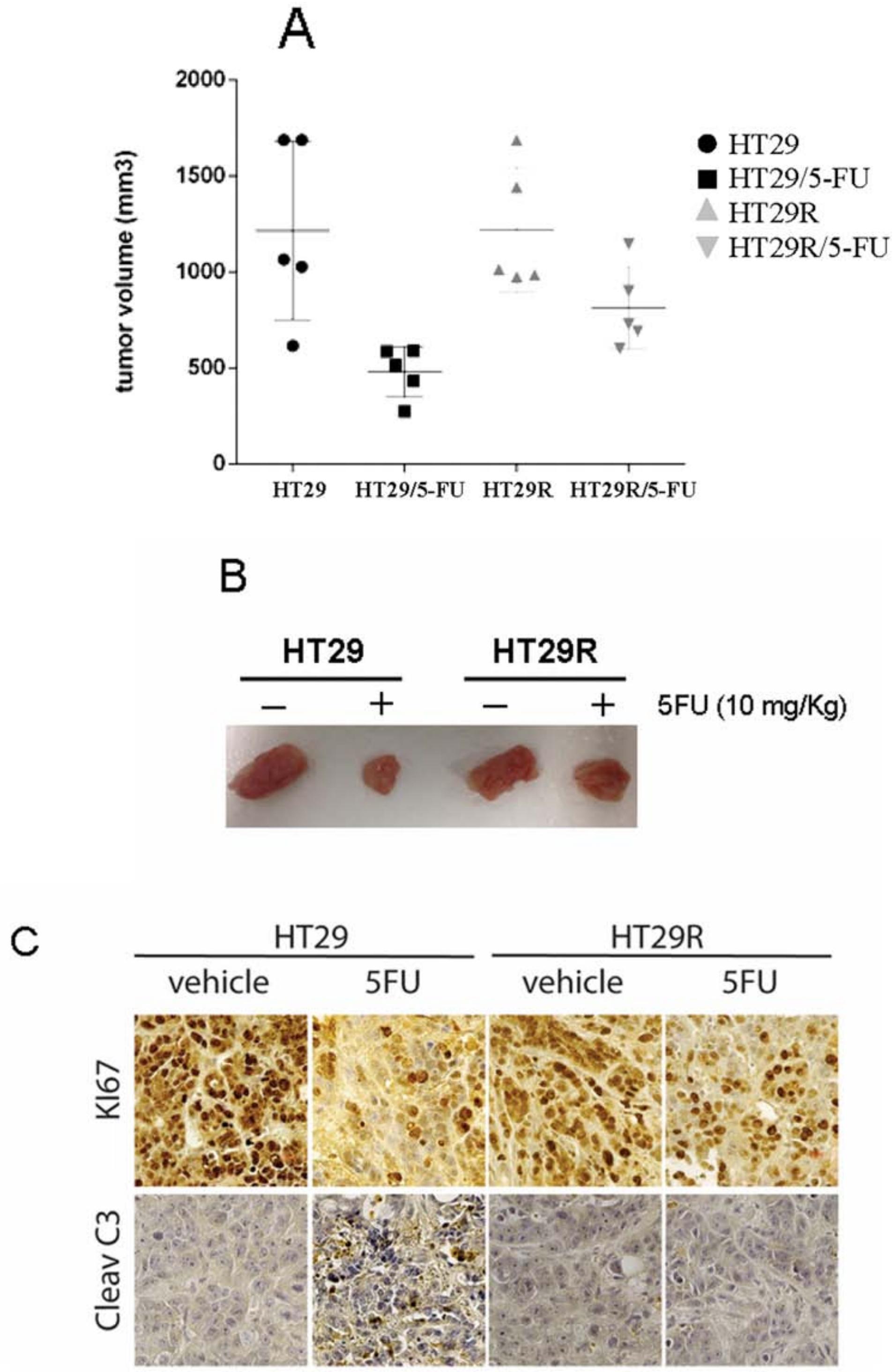

Supplementary Figure S5: To confirm chemoresistance to 5-FU of HT29R cells, *in vivo* xenografts from both sensible and resistant cells were established, mice randomized and daily treated with 10 mg/kg 5-FU. A. Measurement of tumor mass before euthanasia. Data derived from five independent experiments are presented as mean values +/− SD (HT29 Vs HT29R/5-FU, $p = 0.047$; HT29 Vs HT29R, $p = 0.14$; HT29R Vs HT29R/5-FU, $p = 0.83$; HT29/5-FU Vs HT29R/5-FU, $p = 0.017$; HT29/5-FU Vs HT29, $p < 0.01$); **B.** representative images of tumors evidence robust reduction of tumor volumes in both HT29 and HT29R treated groups. **C.** Representative images of immunohistochemical analyses showing that, in HT29 xenograft bearing mice treated with 5-FU, the reduction of tumor volumes is related to drastic reduction of proliferation and induction of apoptosis as suggested by analysis of KI67 and cleaved caspase 3, while, in HT29R-derived tumors, 5-FU treatment caused minimal effect dependent only by a slight reduction of proliferation and no evidence of apoptosis. Scale bar 100 µm.

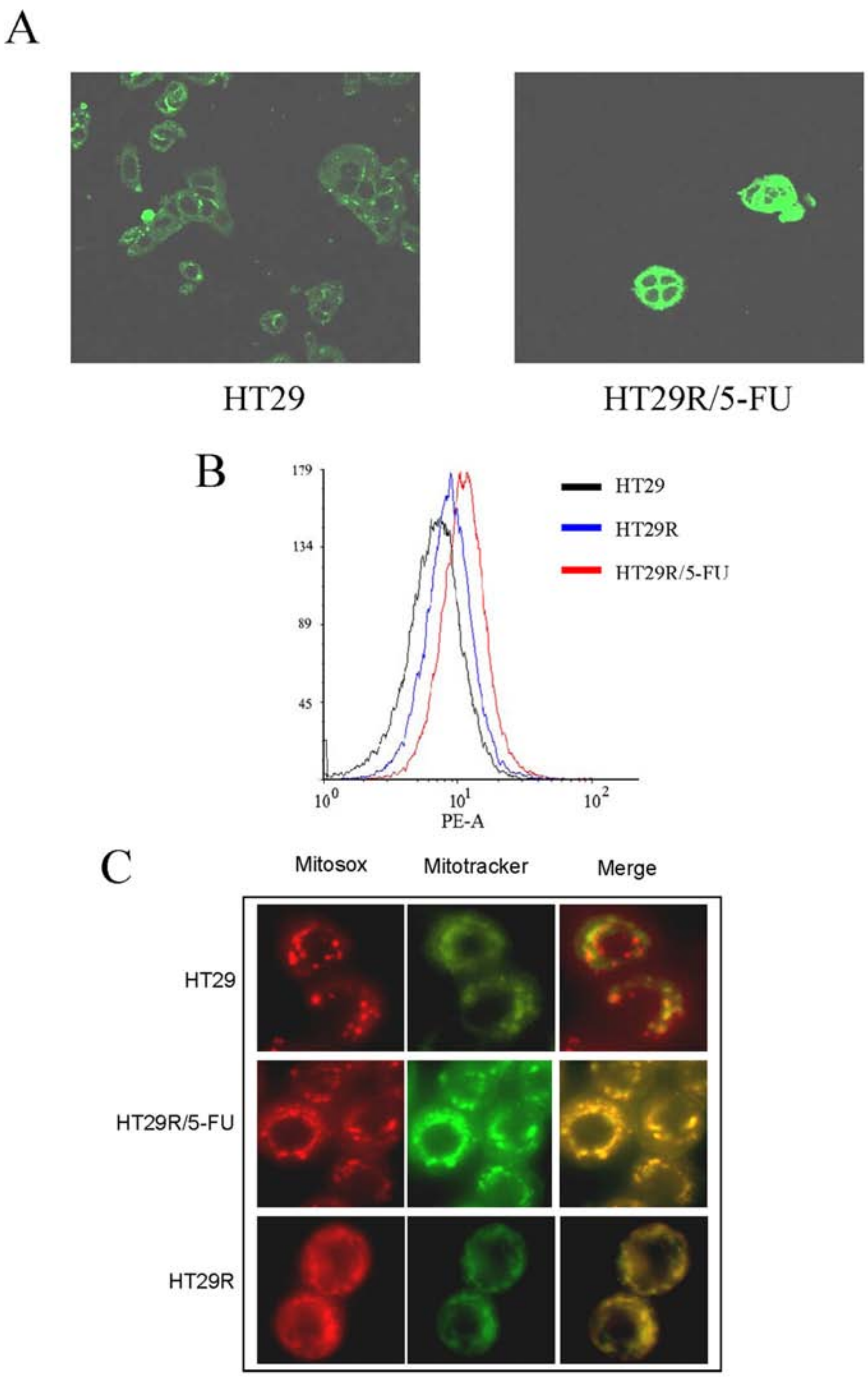

Supplementary Figure S6: A. ROS production of HT29 cells measured using DCF-DA probe. HT29, HT29R/5-FU and HT29R cells (1×10^5) were plated on coverslips inside a Petri dish filled with culture medium After 72 hours growth, cells were incubated for 30 minutes in the presence of the hydrogen peroxide sensitive fluorescent probe (DCF-DA). Samples were then analysed using a confocal microscope. Scale bar 100 µm. **B.** Quantification of mitochondrial ROS was carried out staining cancer cells with Mitosox (500 nM) for 30 minutes. Analyses of samples were carried out using LSRFortessa (BD Bioscience). All experiments were carried out in triplicate. **C.** Detection of mitochondrial ROS as in B. HT29, HT29R/5-FU and HT29R cells (1×10^5) were plated on coverslips inside a Petri dish filled with culture medium. After 72 hours, growth medium was removed, replaced with medium containing Mitosox (500 nM) and Mitotraker (500 nM) probes. Samples were incubated for 20 min, washed with PBS and analysed by fluorescence microscope Nikon Eclipse Ni, equipped with a digital camera (Hamamatsu Digital Camera C11440). Scale bar 20 µm.

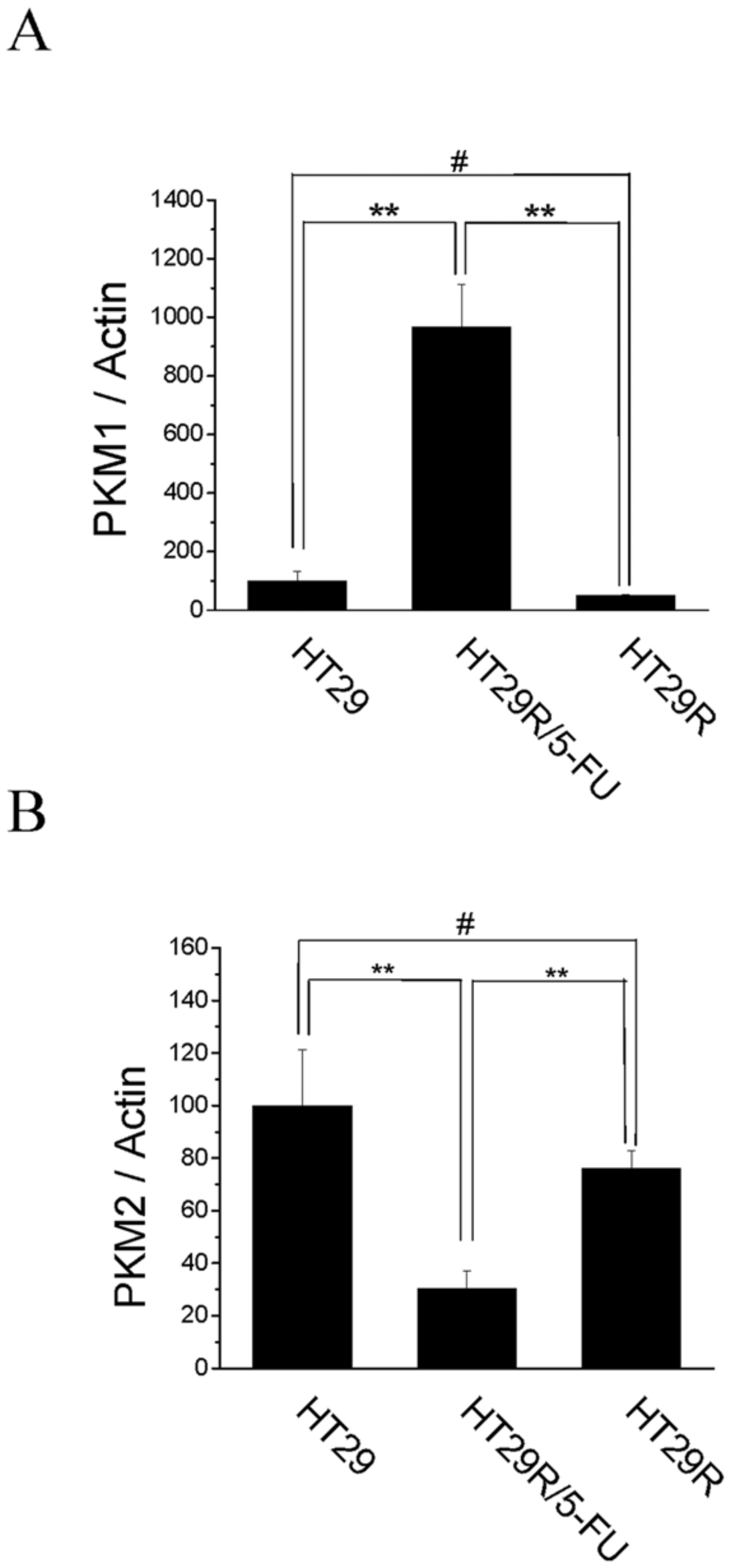

Supplementary Figure S7: Quantification of PKM 1 and PKM2 expression levels. Western blots were probed using specific antibodies able to discriminate the PKM isoforms, conjugated with horse peroxidase (Cell Signaling Technology). Detection of protein was carried out using Clarity ECL Western Blot assay kit. Quantification of immunoblot was carried out using Kodak MI software. All test was carried out in triplicate. Data reported in the figure represent mean value +/− S.E.M.(**$p < 0.01$).

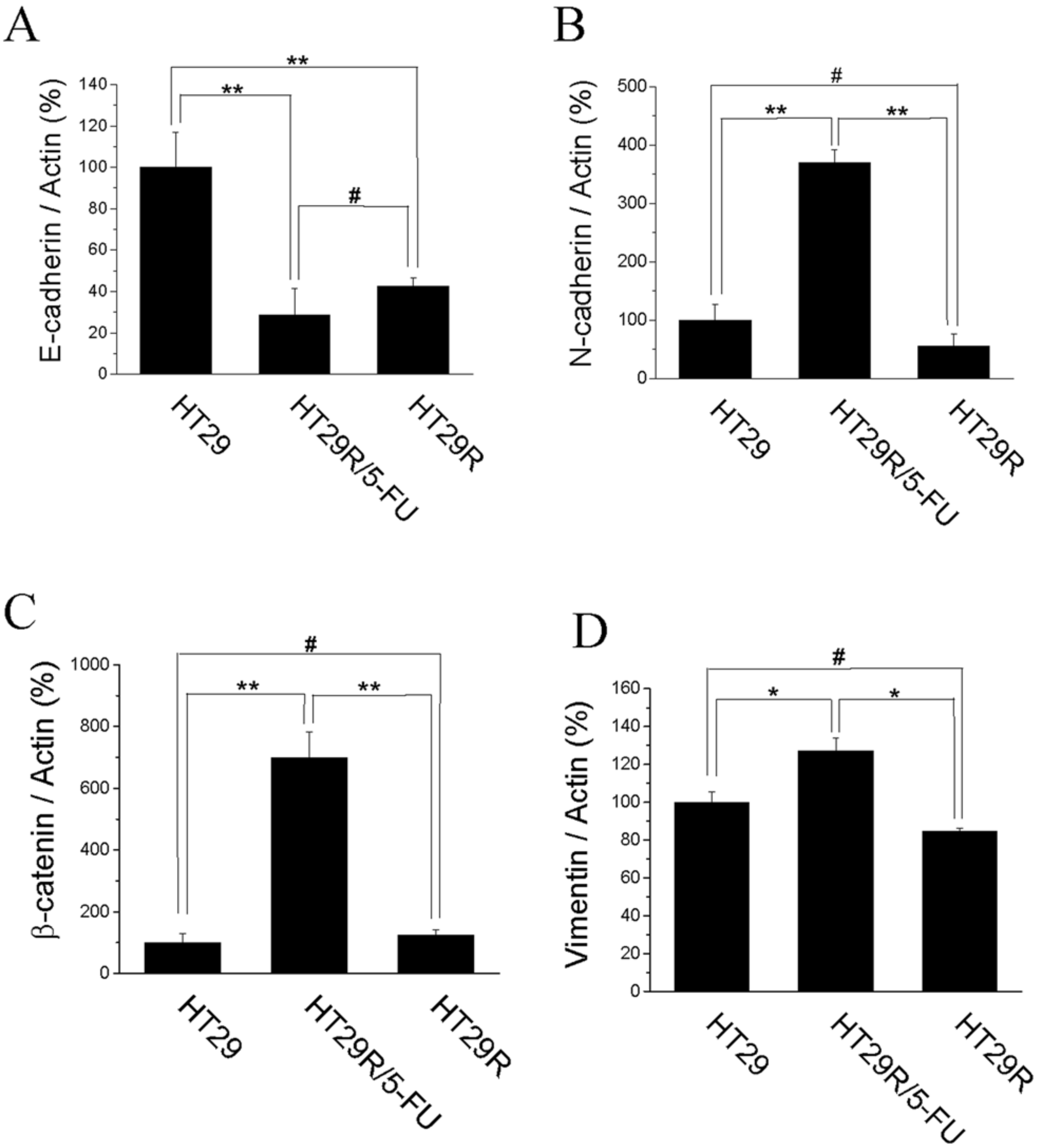

Supplementary Figure S8: Immunoblot quantification of EMT markers. **A.** E-Cadherin; **B.** N-Cadherin; **C.** β-catenin; **D.** Vimentin. Data reported in all plots, represent the mean +/− S.E.M.; each experiment was carried out in triplicate (**$p < 0.01$; *$p < 0.05$).

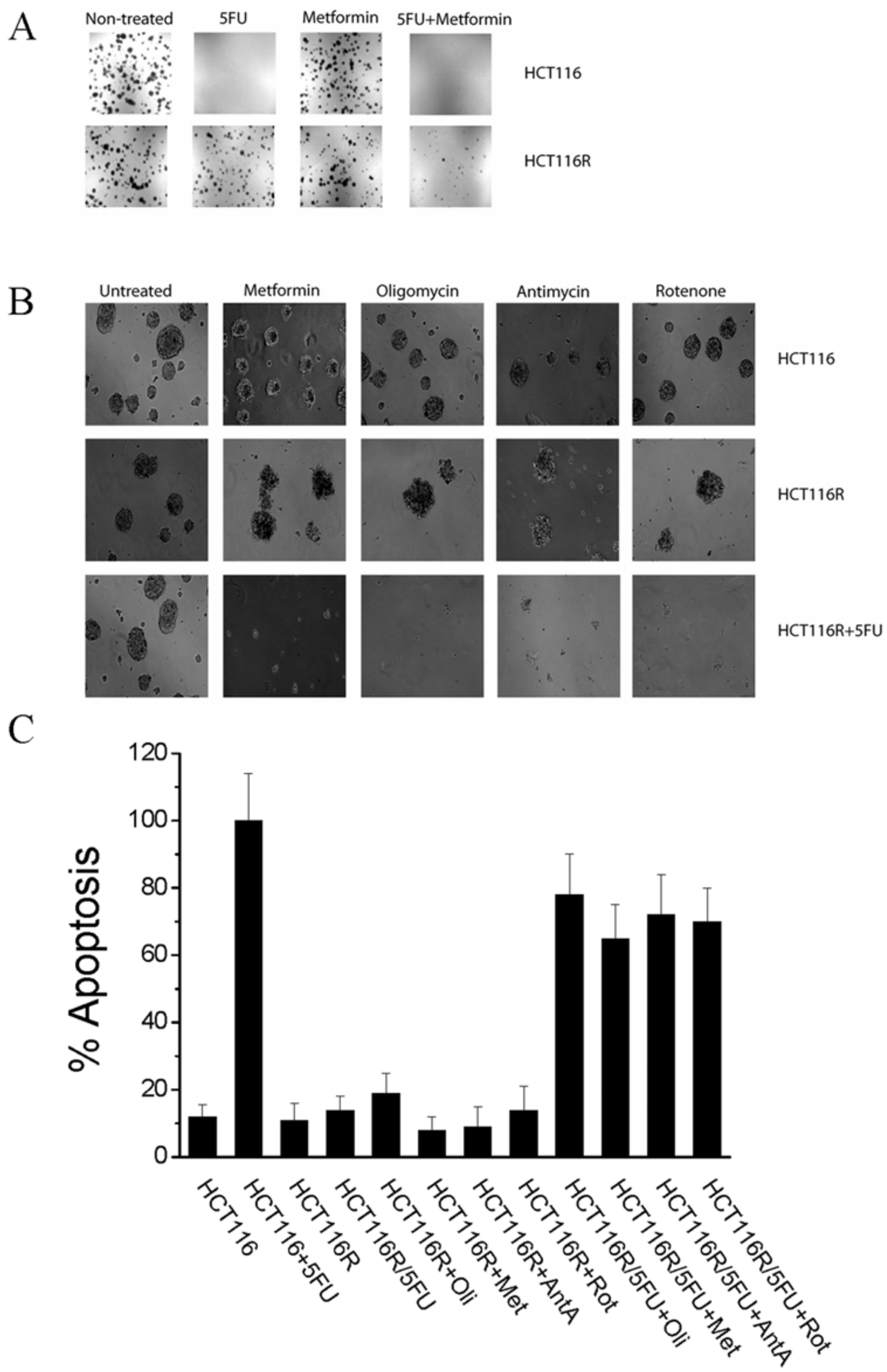

Supplementary Figure S9: To decipher the addiction to respiratory metabolism in HCT116 resistant cells, the effect of combined treatment of 5-FU and different OXPHOS inhibitors was analysed through. A. colony forming Assay of HCT116, HCT116R and HCT116R/5-FU treated with either 20 µM 5-FU, 5 mM Metformin or their combination. Moreover **B.** Anchorage-independent colony forming assay; scale bar 200 µm. **C.** cytofluorimetric quantification of apoptotis of HCT116 and HCT116R treated with either 20 µM 5-FU, different OXPHOS inhibitors or their combination with 5-FU, was carried out showing massive cytotoxic effects on resistant cells.

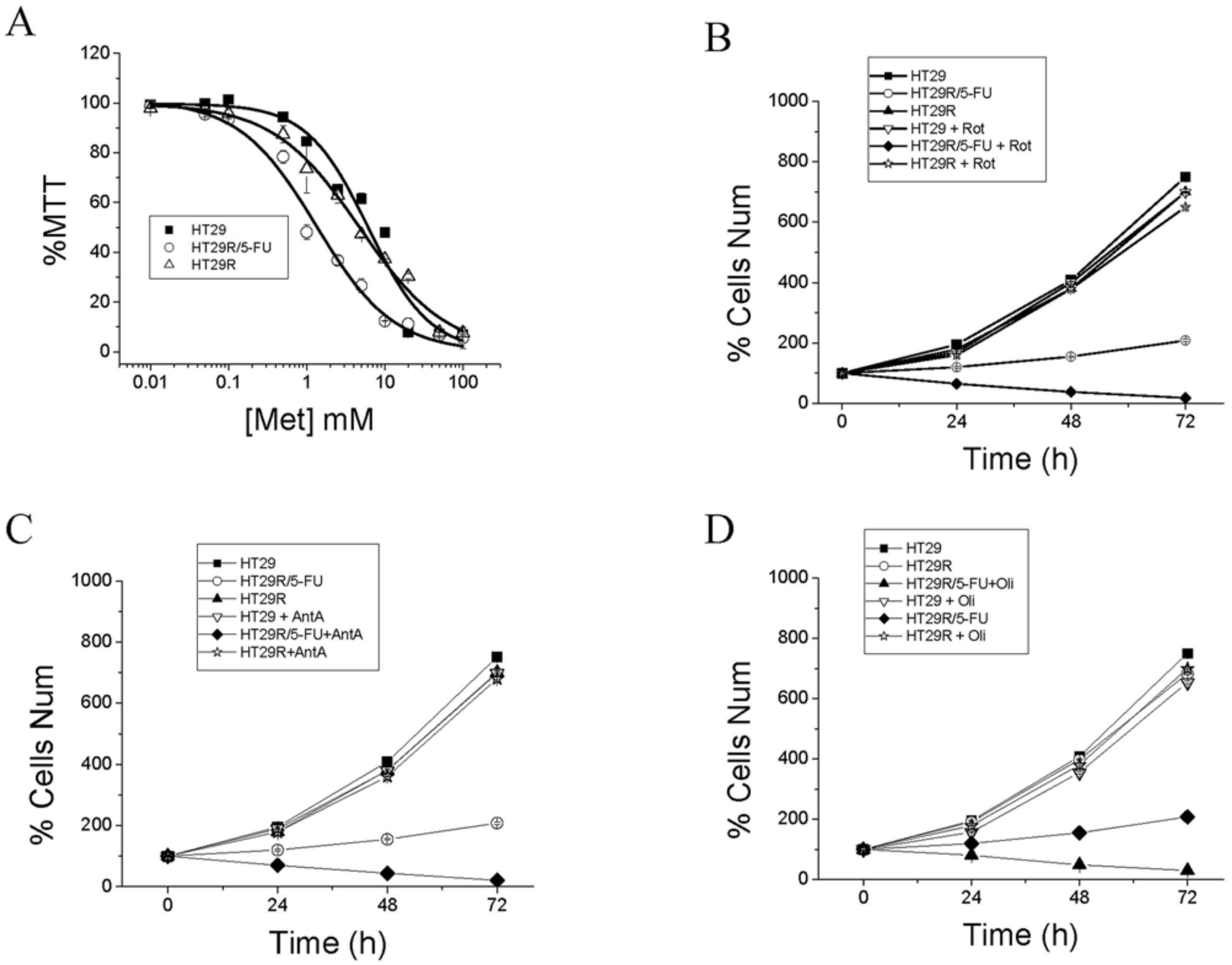

Supplementary Figure S10: A. Metformin IC50 of HT29, HT29R and HT29R cultured in presence of 20 µM 5-FU. The results show that HT29 and HT29R are more resistant to Metformin than HT29R cells acutely treated with 5-FU, with IC50 of: 5.2 ± 0.4 mM (HT29), 4.1 ± 0.3 (HT29R), mM and 1.3 ± 0.1 mM (HT29R/5-FU). **B.** Growth rate of HT29, HT29R and HT29R/5-FU cells in the presence or absence of 1 µM Rotenone, **C.** 1 µM Antimycin A, **D.** 500 nM Oligomycin. The results indicate that only HT29R cells acutely treated with 5-FU show sensitivity to ETC inhibitors, confirming the addiction of these cells to respiratory behaviour induced by 5-FU treatment. Values reported represent the mean value ± S.E.M. The experiments were carried out in triplicate.
